# Application of quantitative proteomics to the integrated analysis of the ubiquitylated and global proteomes of xenograft tumor tissues

**DOI:** 10.1186/s12014-015-9086-5

**Published:** 2015-05-20

**Authors:** Stefani N Thomas, Hui Zhang, Robert J Cotter

**Affiliations:** Department of Pathology, Clinical Chemistry Division, Johns Hopkins University School of Medicine, 1550 Orleans Street CRBII Room 3M06, Baltimore, MD 21231 USA; Department of Pharmacology and Molecular Sciences, Johns Hopkins University School of Medicine, Baltimore, MD 21205 USA

**Keywords:** Ubiquitylation, Mass spectrometry, Proteomics, iTRAQ, Quantification, Xenograft, Tissue

## Abstract

**Background:**

Post-translational modification by ubiquitin is a fundamental regulatory mechanism that is implicated in many cellular processes including the cell cycle, apoptosis, cell adhesion, angiogenesis, and tumor growth. The low stoichiometry of ubiquitylation presents an analytical challenge for the detection of endogenously modified proteins in the absence of enrichment strategies. The recent availability of antibodies recognizing peptides with Lys residues containing a di-Gly ubiquitin remnant (K-ε-GG) has greatly improved the ability to enrich and identify ubiquitylation sites from complex protein lysates via mass spectrometry. To date, there have not been any published studies that quantitatively assess the changes in endogenous ubiquitin-modification protein stoichiometry status at the proteome level from different tissues.

**Results:**

In this study, we applied an integrated quantitative mass spectrometry based approach using isobaric tags for relative and absolute quantitation (iTRAQ) to interrogate the ubiquitin-modified proteome and the cognate global proteome levels from luminal and basal breast cancer patient-derived xenograft tissues. Among the proteins with quantitative global and ubiquitylation data, 91 % had unchanged levels of total protein relative abundance, and less than 5 % of these proteins had up- or down-regulated ubiquitylation levels. Of particular note, greater than half of the proteins with observed changes in their total protein level also had up- or down-regulated changes in their ubiquitylation level.

**Conclusions:**

This is the first report of the application of iTRAQ-based quantification to the integrated analysis of the ubiquitylated and global proteomes at the tissue level. Our results underscore the importance of conducting integrated analyses of the global and ubiquitylated proteomes toward elucidating the specific functional significance of ubiquitylation.

**Electronic supplementary material:**

The online version of this article (doi:10.1186/s12014-015-9086-5) contains supplementary material, which is available to authorized users.

## Background

Ubiquitin is a 76 amino acid, 8.5 kDa protein that is highly conserved among eukaryotes [1–4]. Correspondingly, covalent post-translational modification (PTM) by ubiquitin entails the formation of an isopeptide bond between the C-terminus of ubiquitin and the ε-amino group of the targeted Lys residue on the substrate protein, although it is possible, but rare, for N-terminal amines or Cys residues to be ubiquitylated as well [5]. Protein substrates can be mono-ubiquitylated at one or more Lys residues or they can be poly-ubiquitylated by multiple ubiquitin chains whereby each of the seven Lys residues of ubiquitin can serve as a base to initiate chain formation. Ubiquitylation regulates the localization, activity, and stability of protein substrates [3, 6] with functional consequences related to protein turnover, endocytosis, immune response, transcription, and DNA repair [7, 8].

Ubiquitylation has been implicated in the development and progression of cancer and neurodegenerative diseases [9, 10], and pharmacological inhibitors have been developed that target the ubiquitin-proteasome system for the treatment of cancer [11–13]. In the context of cancer, ubiquitylation regulates tumor-suppressing and tumor-promoting pathways. Thus, the precise analysis of ubiquitylation is required for the identification of ubiquitylated substrates and components of the ubiquitylation pathway that can serve as novel drug targets in the development of highly selective therapeutic compounds. For example, a recent ubiquitylome study by Theurillat *et al*. analyzed changes in the ubiquitin landscape induced by prostate cancer-associated mutations of an E3 ubiquitin ligase substrate-binding protein, thus providing a framework to elucidate the tumorigenic mechanisms linked to dysregulated ubiquitylation [14].

It has only been recently that advances in mass spectrometry and the peptide-based affinity enrichment of the ubiquitylated proteome have permitted the large-scale mapping of ubiquitylation sites [15–22]. The peptide-based affinity enrichment of the ubiquitylated proteome entails the use of a K-ε-GG antibody that specifically recognizes the di-Gly ubiquitin remnant that remains conjugated to Lys residues on substrate proteins following trypsin digestion [16, 22, 23]. K-ε-GG antibodies do not distinguish between ubiquitin and two ubiquitin-like proteins (UBLs) – NEDD8 and ISG15 – that contain C-terminal di-Gly motifs that are generated by trypsin cleavage [16]. However, for the current study, the di-Gly-modified proteome and K-ε-GG-containing peptides will be referred to as the ubiquitylated proteome and ubiquitylated peptides, respectively.

The majority of the published studies that entail the quantitative mass spectrometry-based analysis of ubiquitylation using anti-K-ε-GG antibodies have been conducted using cell lysate [16–20]. Although a study was recently published that quantified the ubiquitin-modified proteome regulated by transient forebrain ischemia in mouse brain tissue using a label-free quantification method [24], to date, there are no published studies of a quantitative analysis to determine the changes in endogenous protein ubiquitylation relative stoichiometry at the tissue proteome level.

The analysis of the protein ubiquitylation level can yield relevant biological insights that cannot be determined from the ubiquitylation or global protein level alone. Prabakaran *et al*. proposed the concept of reversible PTM as a form of information processing [25]. Because PTM substrate molecules can be either modified or unmodified, and the entire pool of substrate molecules is comprised of a mixture of both molecular states, the relative stoichiometry of the modified state is therefore representative of the state of the population of the substrate molecule. Olsen *et al*. illustrated this concept in the context of phosphorylation site occupancy during mitosis [26].

The relative stoichiometry of the modified state of the population of substrate molecules is a function of the PTM’s “writers” and “erasers” [27]. Ubiquitylation entails a highly regulated enzymatic cascade involving ubiquitin activating (E1), ubiquitin conjugating (E2), and ubiquitin ligating (E3) enzymes [6]. In addition to the E1, E2, and E3 enzymes that are considered “writers” of ubiquitylation, approximately 100 ubiquitylation “erasers” exist in the form of deubiquitylating enzymes [28]. Hence, complex enzymatic cascades regulate ubiquitylation.

Analyzing differences in relative abundance that are specifically attributable to ubiquitylation instead of the global protein level is an important step towards the determination of the functional relevance of ubiquitylation in the proteome of interest. Accordingly, in this study, we used a mass spectrometry-based quantitative proteomic approach combined with the immunoaffinity enrichment of di-Gly-modified peptides to quantitatively analyze the relative stoichiometry of protein ubiquitylation in breast cancer patient-derived xenograft tissues using iTRAQ. We used basal and luminal human-in-mouse breast cancer patient-derived xenografts as two disparate tumor types from which we would expect to observe quantitative differences in global protein levels and ubiquitylation levels. Our integrated approach permitted the analysis of changes in relative abundance not only at the ubiquitylated protein level, but also at the global protein level. To our knowledge, this study is the first integrated quantitative mass spectrometry-based analysis of endogenous ubiquitylation relative stoichiometry in tissues.

## Results and discussion

### Quantitative approach to interrogate the ubiquitylated proteome of tissues

The goal of this study was to identify up- or down-regulated ubiquitylation sites on proteins with stable global relative abundance in basal and luminal breast cancer patient-derived xenograft tissue toward the characterization of ubiquitylation sites that could be involved in biological information processing. The xenografts were derived from breast cancer patients with poor prognosis and treatment-resistant disease [29]. Following protein extraction and trypsin digestion, peptides from each breast cancer patient-derived xenograft tumor tissue sample (two basal and two luminal xenograft tissue aliquots) were subjected to di-Gly ubiquitin remnant motif (K-ε-GG) immunoaffinity enrichment using an antibody that recognizes Lys residues that are modified with a di-Gly ubiquitin remnant (K-ε-GG).

Chemical stable isotope labeling using iTRAQ was applied to enable the relative quantification of ubiquitylation between the samples. However, because the K-ε-GG-specific antibody only recognizes unlabeled K-ε-GG-peptides, the iTRAQ labeling of the ubiquitylated proteome was conducted after K-ε-GG enrichment. The peptides were labeled with iTRAQ-114 (basal xenograft 1), iTRAQ-115 (basal xenograft 2), iTRAQ-116 (luminal xenograft 1), and iTRAQ-117 (luminal xenograft 2) reagents from a 4-plex iTRAQ reagent kit, and they were subjected to LC-MS/MS analysis.

Because the primary amine group of the di-Gly ubiquitin remnant on the target Lys of the substrate protein can be modified by iTRAQ reagents, in addition to the constant and variable modifications that were used for peptide and protein identification for the global proteome analysis, variable modifications of +258.1449 Da (mass of iTRAQ 4-plex reagent added to di-Gly ubiquitin remnant) and +114.0429 Da (mass of di-Gly ubiquitin remnant) were added to Lys residues when conducting database searches of the ubiquitylated proteome data. Another consideration for the analysis of the ubiquitylated proteome data is that results from large-scale analyses of ubiquitin modified proteomes have indicated that trypsin, which has a strict sequence specificity [30], does not cleave at di-Gly-modified Lys residues [20, 21]. Hence, missed cleavage at di-Gly-modified Lys residues provides confidence for ubiquitylation site localization [31].

The enrichment selectivity of the ubiquitylated peptides was 80 %, as determined by the number of peptide-spectrum matches (PSMs) of ubiquitylated peptides (357) divided by the total number of PSMs of all identified peptides (450). In the absence of immunoaffinity enrichment, 0.02 % of the identified peptides were ubiquitylated, and only 2 ubiquitylated peptides were identified in common with the enriched samples: 1 polyubiquitin peptide with 4 PSMs, and 1 histone H2A.J peptide with 10 PSMs (data not shown). The remaining 8 ubiquitylated peptides identified in the global dataset were identified by 1 PSM. The very low yield of ubiquitylated peptides in the non-enriched samples supports the need for an enrichment step in the mass spectrometry-based analysis of the ubiquitylated proteome of complex biological samples.

In the enriched dataset, the majority (99.7 %) of the ubiquitylated peptides had a charge ≥3+, which reflects the additional charge from the N-terminal amine on the di-Gly adduct from the C-terminal tryptic ubiquitin remnant. The average peptide yield after immunoaffinity enrichment was 5 μg from an input of 3 mg of peptides.

**Fig. 1:**
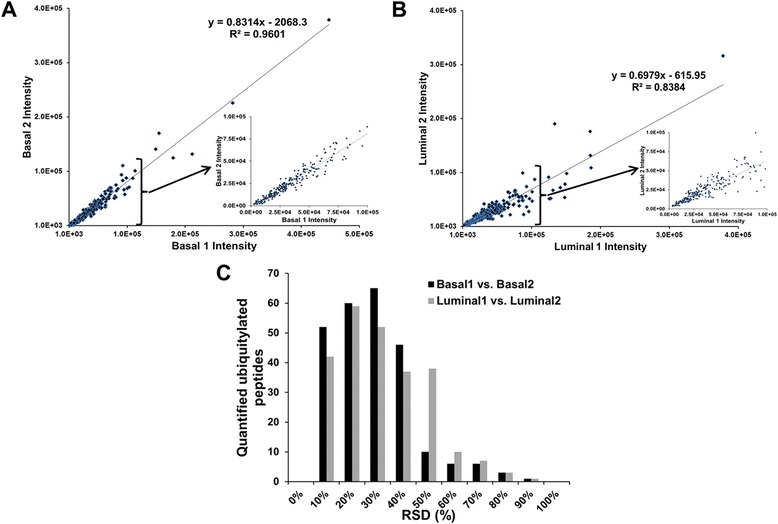
Reproducibility of iTRAQ-based quantitative analysis of ubiquitylation in basal and luminal breast tumor xenografts. **a** iTRAQ reporter ion intensities of ubiquitylated peptides from the Basal 1 vs. Basal 2 xenografts. The inset shows an enlarged region of the plot for the ubiquitylated peptides with intensities <1.0e^5^. **b** iTRAQ reporter ion intensities of ubiquitylated peptides from the Luminal 1 vs. Luminal 2 xenografts. The inset shows an enlarged region of the plot for the ubiquitylated peptides with intensities <1.0e^5^. **c** Distribution of RSDs (%) of the iTRAQ reporter ion intensities of ubiquitylated peptides from Basal 1 vs. Basal 2 (black bars) and Luminal 1 vs. Luminal 2 (grey bars) xenografts

To evaluate the reproducibility of the quantified ubiquitylated peptides of human origin in the luminal vs. basal breast tumor xenograft tissues, a linear regression analysis was conducted on the correlation of the iTRAQ reporter ion intensities in each luminal and basal xenograft tissue specimen. Ideally, the iTRAQ reporter ion intensities associated with the same peptide from both replicates would be nearly identical. The reproducibility of the two replicates (Basal 1 vs. Basal 2, Fig. 1a and Luminal 1 vs. Luminal 2, Fig. 1b) was assessed based on the slope and R^2^ values of the linear regressions. The slope of the linear regression fit to the basal xenograft replicates was 0.8314, whereas the slope of the linear regression fit to the luminal xenograft replicates was 0.6979. The 0.9601 and 0.8384 R^2^ values of the linear regressions of the basal and luminal replicates, respectively, indicate acceptable reproducibility. Technical variability was further assessed by the distribution of the RSDs of the log_2_-transformed iTRAQ reporter ion intensities of the quantified ubiquitylated peptides (Fig. 1c). The mean iTRAQ reporter ion intensity RSDs of the basal and luminal replicates were 23 % and 27 %, respectively, which indicates the degree of technical variability of the workflow that we developed to quantify endogenous ubiquitylation in tissue samples.

**Fig. 2:**
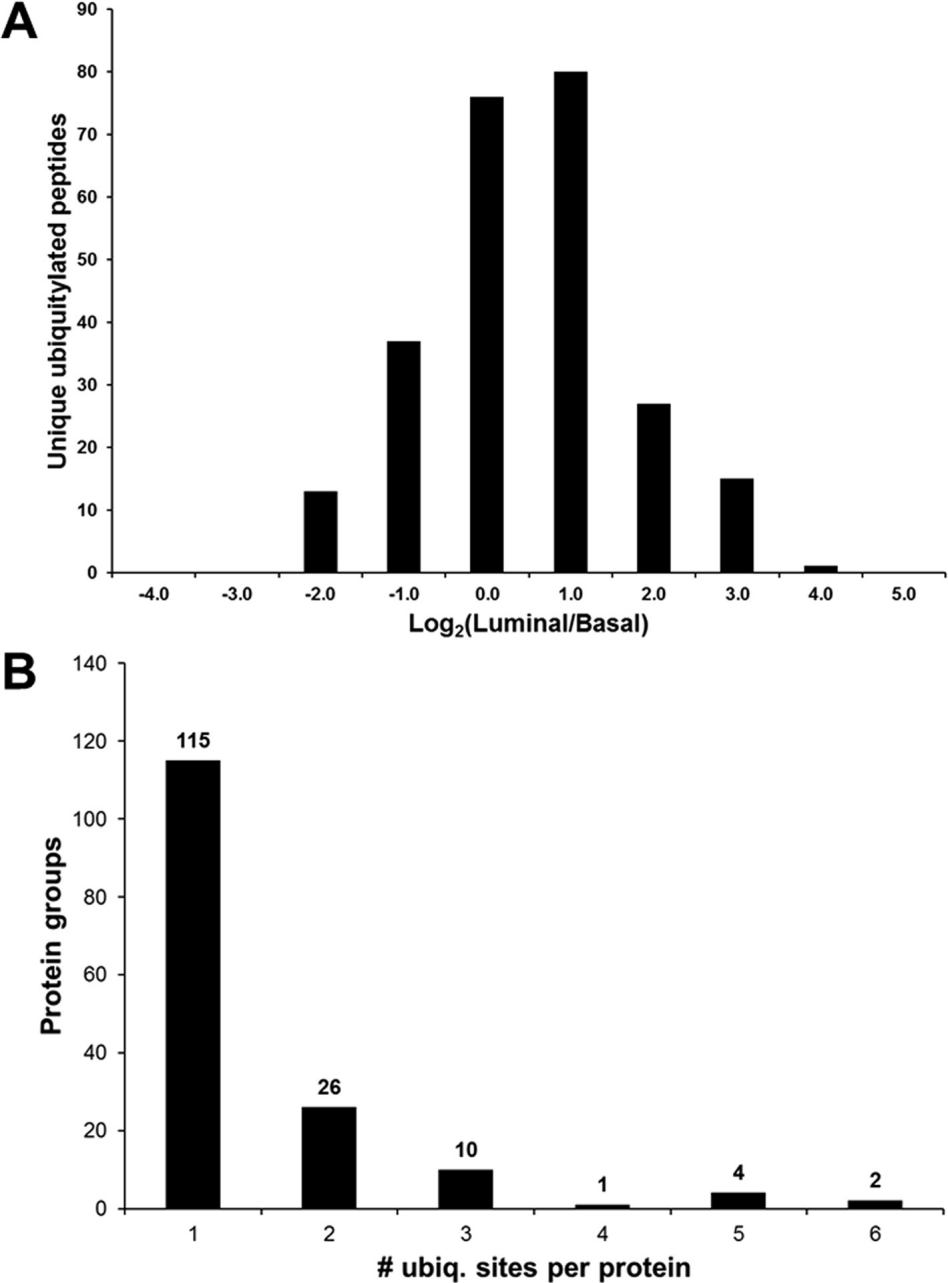
Distribution of the quantified ubiquitylated peptides of human origin. **a** A total of 249 ubiquitylated peptides of human origin were quantified with a mean relative abundance of -0.004. For each ubiquitylated peptide, the average iTRAQ reporter ion intensity values of the luminal samples (116 and 117 labels) were divided by the average iTRAQ reporter ion intensity values of the basal samples (114 and 115) and log_2_-transformed. **b** Number of ubiquitylated proteins vs. the number of ubiquitylation sites per protein

This iTRAQ-based quantitative proteomic approach to analyze the ubiquitin-modified proteome of luminal and basal breast tumor xenografts resulted in the identification of 327 unique ubiquitylated peptides at a 1 % false discovery rate and 319 unique non-ambiguously localized ubiquitylation sites that were quantified across all four samples (Additional file 1: Table S1). For each ubiquitylated peptide, the average iTRAQ reporter ion intensity values of the luminal samples (116 and 117 labels) were divided by the average iTRAQ reporter ion intensity values of the basal samples (114 and 115) and log_2_-transformed. The mean ratio of these peptides was -0.004 (log_2_(luminal/basal)) with a standard error of 0.06 indicating that the data are approximately normally distributed. The distribution of the relative abundance ratios of the ubiquitylated peptides is shown in Fig. 2a.

Reflecting the mixed human and murine proteome backgrounds of the xenograft samples, 76 % of the identified unique ubiquitylated peptides (249) were derived from 173 proteins of human origin. The identified ubiquitin-derived poly-ubiquitylated peptides were common to *Mus musculus* polyubiquitin and *Homo sapiens* ubiquitin-40S ribosomal protein S27a; therefore, it could not be definitively determined whether these 6 peptides were of human or murine origin. Proteins with functions related to the ubiquitylation machinery (E2 ubiquitin conjugating enzymes, E3 ubiquitin ligases, and proteasome subunits) and ubiquitin-like modifiers (NEDD8 and SUMO 2) were among the quantified ubiquitylated proteins.

Whereas the majority of the ubiquitylated proteins contained only 1 ubiquitylation site (115 proteins), 43 proteins contained >1 ubiquitylation site including 4 proteins that had 5 ubiquitylation sites and 2 proteins that had 6 ubiquitylation sites (Fig. 2b). Of the 43 proteins containing multiple ubiquitylation sites, 6 contained ubiquitylation sites that did not exhibit the same trend in relative abundance between the basal and luminal xenografts. For these proteins, some sites had higher levels of relative abundance in the basal samples, whereas other sites in the same protein had higher levels of relative abundance in the luminal samples. This result is suggestive of the well-known function of ubiquitylation in conferring site-specific differential modes of regulation on substrate proteins [2].

Ubiquitin was among the quantified ubiquitylated proteins. Six of its seven Lys residues (K6, K27, K29, K33, K48 and K63) (Additional file 1: Table S1) were quantified. These Lys residues are known to form poly-ubiquitin linkages, and the specific Lys residue that is involved in the linkage confers different cellular functions on the substrate proteins. K48 linkages are considered canonical signals for proteasomal degradation by the 26S proteasome [32]; K63 linkages are known to be involved in several non-proteolytic processes such as protein sorting, NF-κB signaling, kinase activation, and translational control [33]; and K6, K27, K29, and K33 linkages are hypothesized to have roles in DNA repair [34]. None of the six quantified ubiquitylation sites were up- or down-regulated, and the global protein level of ubiquitin was stable [average (log_2_(luminal/basal) = −0.03)].

**Fig. 3:**
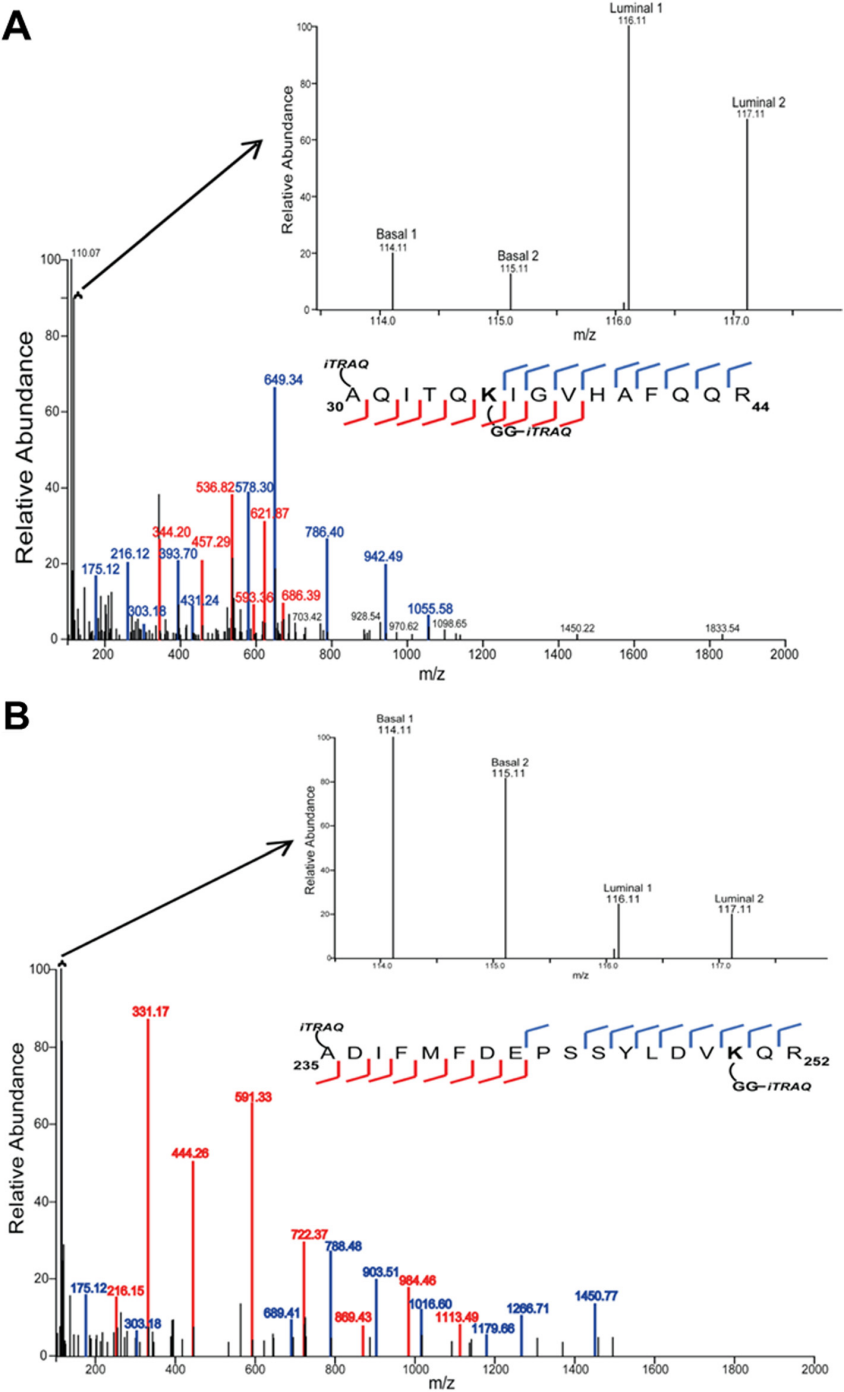
Representative spectra of quantified up-regulated and down-regulated ubiquitylated peptides. **a** Up-regulated ubiquitylated peptide from ubiquitin-like protein ISG15 precursor (aa30-44) with ubiquitylation of K35. The main figure shows the MS/MS spectrum and the corresponding ion coverage of the peptide to which the spectrum was assigned. The inset shows an enlarged region of the MS/MS spectrum (113.0 – 118.0 m/z) with the iTRAQ reporter ions. Based on the intensity of the 114.11 and 115.11 m/z (basal xenografts) and the 116.11 and 117.11 m/z (luminal xenograft) reporter ions, the peptide was determined to be up-regulated 2.69-fold in the luminal compared to the basal tumor sample. **b** Down-regulated ubiquitylated peptide from ATP-binding cassette sub-family E member 1 (aa235-252) with ubiquitylation of K250. The main figure shows the MS/MS spectrum and the corresponding ion coverage of the peptide to which the spectrum was assigned. The inset shows an enlarged region of the MS/MS spectrum (113.5 – 118.0 m/z) with the iTRAQ reporter ions. The peptide was determined to be down-regulated 2.36-fold in the luminal compared to the basal tumor samples

Representative peptides with up-regulated and down-regulated ubiquitylation sites are presented in Fig. 3. Up-regulated and down-regulated peptides were considered as those with log_2_(luminal/basal) values that were greater or less than the mean ± 2 s.d. of the distribution of the ratios for each dataset. Shown in Fig. 3a is a representative spectrum of an ubiquitylated peptide from ubiquitin-like protein ISG15 precursor with an up-regulated ubiquitylation site (K35) in the luminal compared to the basal tumor xenografts. The di-Gly ubiquitin remnant on K35 was labeled with the iTRAQ reagent, and the relative abundance ratio (log_2_(luminal/basal)) was 2.69. Fig. 3b is a representative MS/MS spectrum of an ubiquitylated peptide from ATP-binding cassette sub-family E member 1 with a down-regulated ubiquitylation site (K250) in the luminal compared to the basal tumor xenografts. The di-Gly ubiquitin remnant on K250 was labeled with the iTRAQ reagent, and the relative abundance ratio (log_2_(luminal/basal)) was −2.36. The cognate unmodified (non ubiquitylated) peptide with enzymatic cleavage occurring at K250 was identified in the global proteome dataset with an average log_2_(luminal/basal) relative abundance ratio of −1.03. Some of the ubiquitylated peptides that were not identified in their cognate unmodified form contained di-Gly-modified Lys residues at locations in the peptide that would result in tryptic peptides with m/z values below the mass spectrometer’s detection range.

**Fig. 4:**
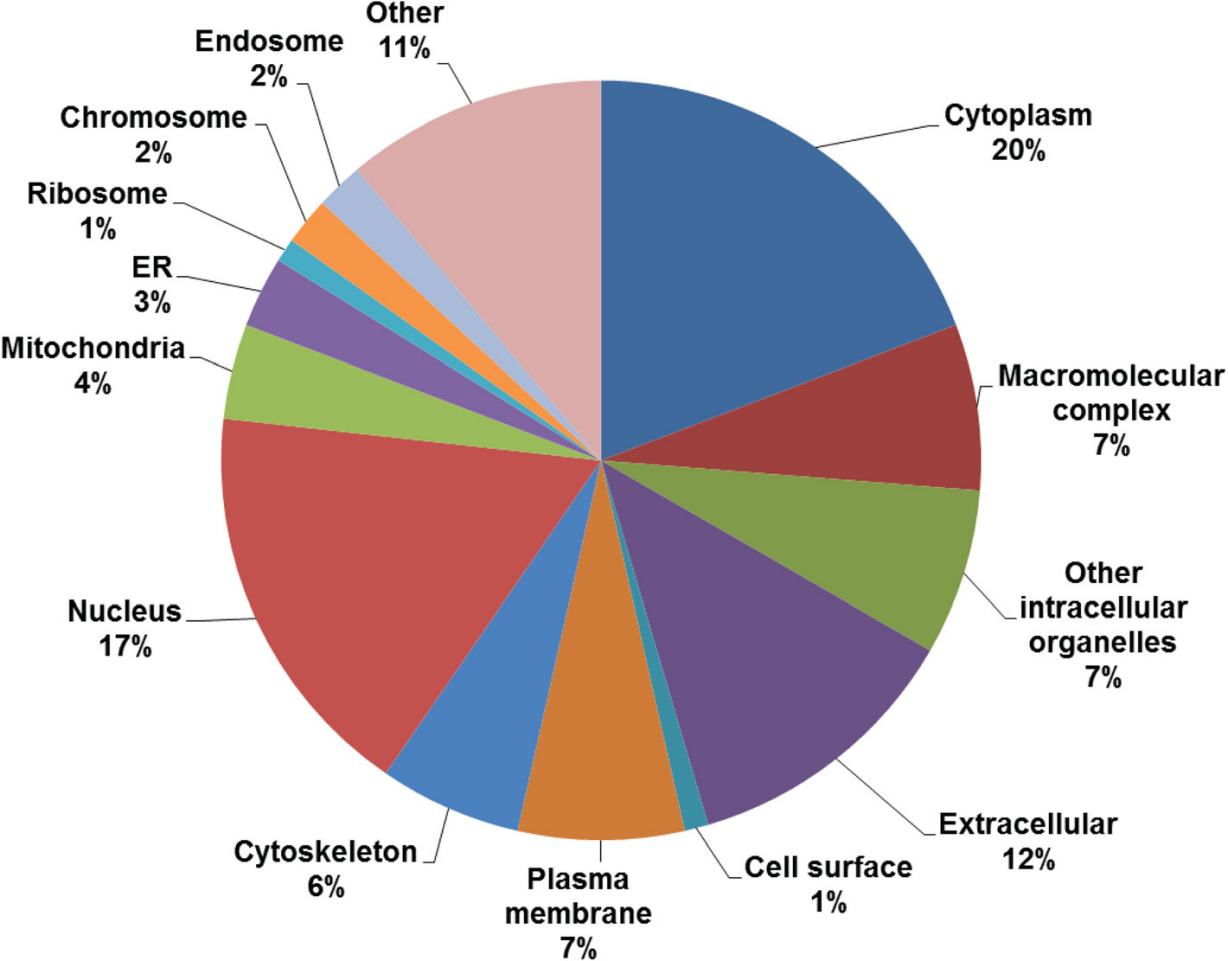
GO cellular component categorization of ubiquitylated proteins

To evaluate the cellular distribution of the ubiquitylated proteins, Gene Ontology (GO) cellular compartment analysis was conducted using STRAP [35]. Proteins of human and murine origin were used for this analysis. The results indicate that the ubiquitylated proteins were present in all major cellular compartments, and there was a nearly equal distribution of ubiquitylated proteins between the cytoplasm (19 %) and nucleus (17 %) (Fig. 4). The cellular component distribution of the ubiquitylated proteins indicates that the enrichment was not biased toward ubiquitylated proteins from a specific region of the cell.

### Global proteome analysis yields the identification and quantification of hormone receptor and tumor antigen proteins commonly used to characterize luminal and basal breast cancer subtypes

The determination of differentially regulated ubiquitylation sites relies on analysis of not only the ubiquitylated proteome, but also the global proteome. To enable the analysis of the basal and luminal tumor xenograft global proteomes, 5 % of each protein digest was chemically labeled with 4-plex iTRAQ reagents 114 and 115 (basal xenografts 1 and 2, respectively) and 116 and 117 (luminal xenografts 1 and 2, respectively). An equivalent amount of peptides from the four samples was combined and subjected to offline bRPLC fractionation. The 96 bRPLC fractions were concatenated in a non-contiguous manner to yield 24 fractions. Concatenating multiple fractions that have minimal overlap and cover a wide separation window improves the effective orthogonality of bRPLC first dimension separation to the second dimension online RPLC separation [36]. These fractions were then subjected to LC-MS/MS analysis, which resulted in a total of 5,416 quantified protein groups with a minimum of two unique peptides. Reflecting the species origin of the samples, 78 % (4,244) were *Homo sapiens* proteins and 22 % (1,172) were *Mus musculus* proteins. A total of 46,393 unique peptides were quantified, among which 46,258 were quantified across all four xenograft tumor tissue samples.

**Fig. 5:**
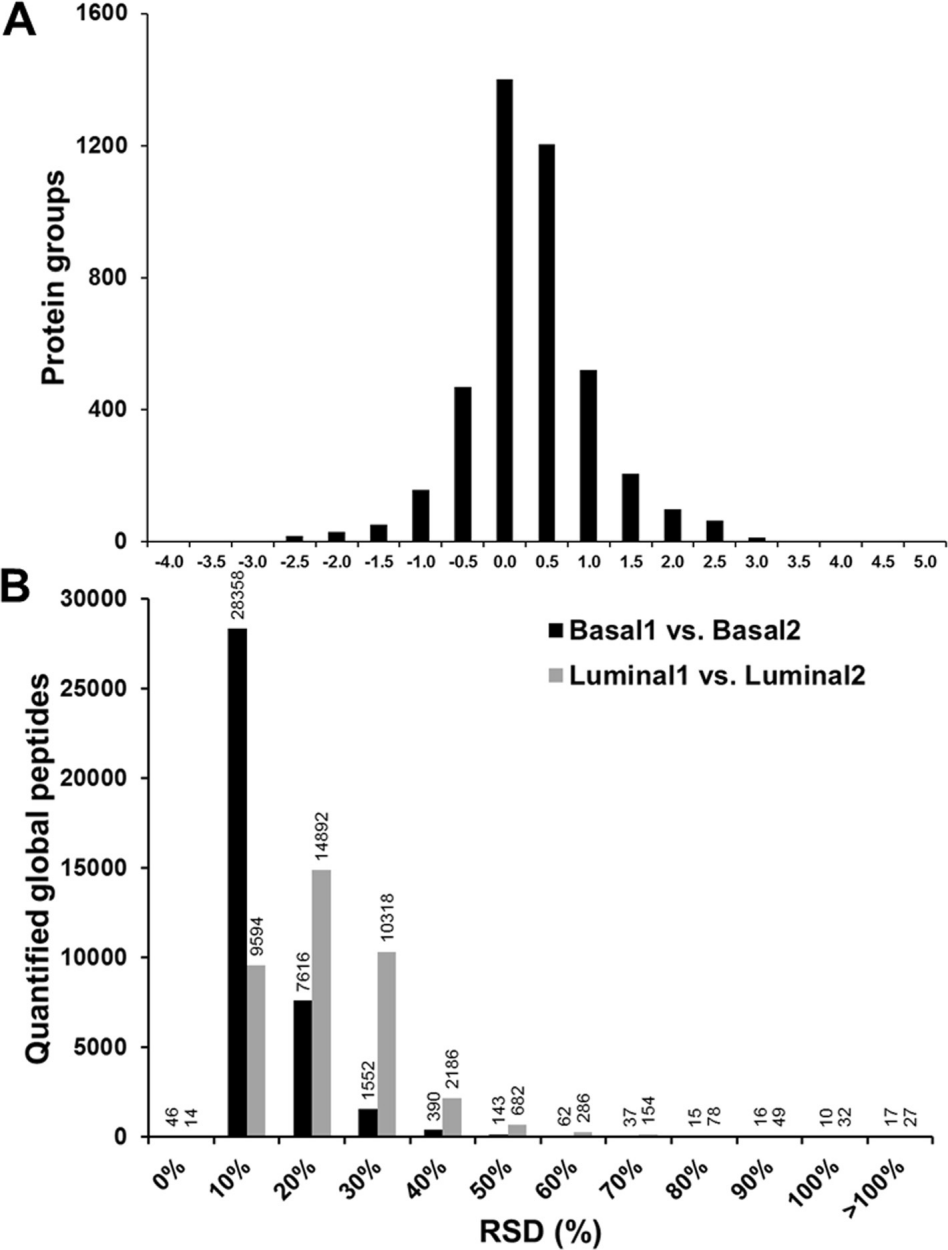
Distribution of the quantified proteins from the global analysis workflow. **a** A total of 4,244 proteins of human origin were quantified with a mean relative abundance ratio of 0.00 ± 0.01, where 0.01 is the standard error of the mean. The ratios are reported as log_2_(luminal/basal). **b** Assessment of technical process replicate reproducibility (Basal1 vs. Basal2, black bars, and Luminal1 vs. Luminal2, grey bars) based on the RSD (%) of the iTRAQ reporter ion intensities of the quantified global peptides

The iTRAQ reporter ion intensity ratios were log_2_-transformed. The mean and standard error of the protein ratios was 0.00 ± 0.01 (Additional file 2: Table S2). The distribution of the quantified proteins of human origin is shown in Fig. 5a. The technical process replicate reproducibility of the global proteome analysis workflow was assessed based on the RSD (%) of the iTRAQ reporter ion intensities of the quantified global peptides (Fig. 5b). The mean RSD of the iTRAQ reporter ion intensities of the quantified global peptides from the Basal samples was 8 %, whereas the mean RSD from the Luminal samples was 18 %. The lower RSDs of the quantified global peptides compared to the ubiquitylated peptides reflect the differences in the workflows for these two proteomes. Unlike the ubiquitylation workflow, the basal and luminal tissue-derived peptides for global analysis were combined immediately following iTRAQ labeling and prior to bRPLC fractionation and LC-MS/MS analysis, whereas the peptides for ubiquitylation analysis were not combined until after digestion, enrichment, and iTRAQ labeling.

Ubiquitin/ISG15 conjugating enzyme was the most abundant protein in the luminal xenografts (log_2_(luminal/basal) = 3.36). Ubiquitin/ISG15 conjugating enzyme is an E2 ubiquitin-conjugating enzyme that functions to couple the activation of ubiquitin to downstream conjugation events, thereby influencing the fate of substrate proteins. Relative abundance is not directly related to enzymatic activity; therefore, the higher relative abundance of Ubiquitin/ISG15 conjugating enzyme in the luminal xenografts does not necessarily indicate an overall elevation of ubiquitylation in these xenografts compared to the basal xenografts. There are 30 members of the E2 ubiquitin-conjugating enzyme family, 15 of which were quantified in this study. Whereas 6 of these E2 enzymes displayed higher levels of relative abundance in the luminal xenografts, 9 exhibited higher levels of relative abundance in the basal xenografts. This result reflects the complexity of ubiquitylation that is partially attributable to the substrate specificity and ubiquitin linkage topologies of the 30 E2 conjugating enzymes and the >1000 E3 ligating enzymes in the ubiquitylation enzymatic cascade.

The depth of the proteome coverage permitted the identification and quantification of proteins that are clinically used to stratify breast cancer subtypes [37]: epidermal growth factor receptor isoform 1 (log_2_(luminal/basal) = −1.04), cellular tumor antigen p53 (log_2_(luminal/basal) = 2.06), membrane-associated progesterone receptor component 1 (log_2_(luminal/basal) = −0.44), and membrane-associated progesterone receptor component 2 (log_2_(luminal/basal) = 0.09). The expression of progesterone receptors (PR) is generally associated with luminal breast cancers (PR+), whereas triple negative or basal-like tumors are typically PR- [37, 38]. The quantification of PR indicates that it was present in the basal and luminal xenografts. Not all basal-like tumors are triple negative, and it has been shown that much of the clinically observable plasticity and heterogeneity occurs within, and not across, the major biological breast cancer subtypes [37–39].

The molecular clinical subtype information for the patients from whom the basal and luminal breast cancer xenograft models were derived indicate that the basal xenograft is HER2-, ER- and PR-, whereas the luminal xenograft (luminal A subtype) is HER2-, ER+ and PR+ [29]. However, these phenotypes were validated at passage 1 (P1) for the xenografts, and the xenografts used for this study were acquired after P32 (basal) and P33 (luminal) following extensive expansion. No actively growing cancer has a static genome, and genetic drift is inherent in the xenografting process. Results from a late-exome expansion study to characterize genomic drift in the basal patient-derived xenograft used in this study indicated the presence of moderate genomic instability determined by the detection of several single nucleotide variations [29]. Furthermore, although the basal and luminal xenografts were clinically characterized as being HER2-, Western blot data indicated low levels of HER2 protein expression [29].

Taken together, the results from our global proteomic analysis indicate that the workflow resulted in an adequate depth of coverage to permit the identification and quantification of hormone receptor and tumor antigen proteins that are typically used to classify breast cancer subtypes.

### Differences in abundance of global proteins vs. ubiquitylated proteins suggest differential ubiquitylation site occupancy

**Fig. 6:**
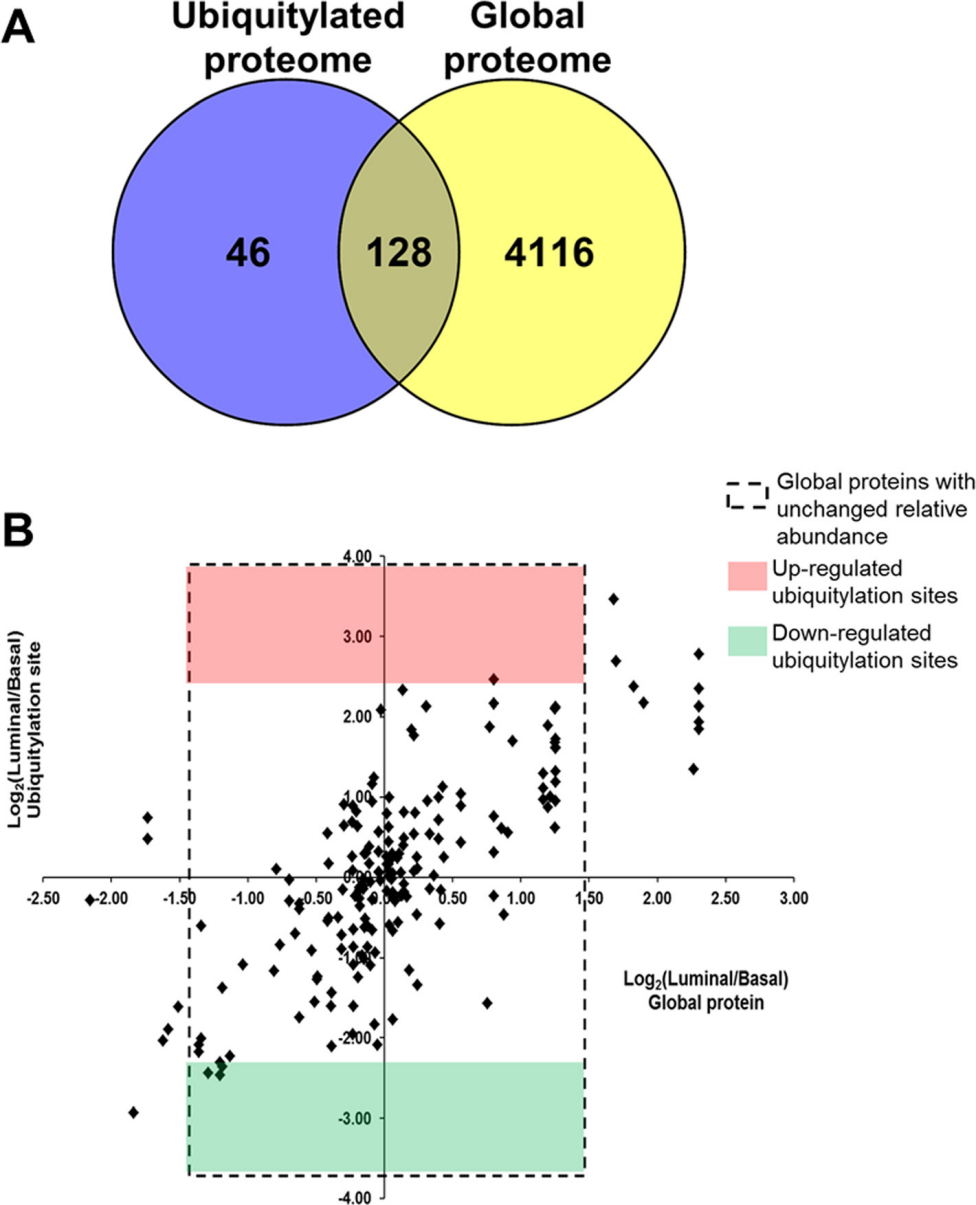
Relative abundance of global proteome vs. ubiquitylated proteome. **a** Among the 4,244 quantified proteins of human origin from the global analysis and the 173 quantified proteins of human origin from the ubiquitylation analysis, 128 proteins were identified in common. **b** Among the 128 commonly identified proteins, 116 had unchanged total relative abundance levels (dashed rectangular box). Of these 116 proteins, 1 had up-regulated ubiquitylation levels in the luminal xenografts (red box) and 3 had down-regulated ubiquitylation levels in the luminal xenografts (green box)

After analyzing the global and ubiquitylated proteomes of the basal and luminal breast tumor xenografts, we sought to analyze differences in relative abundance between the global protein levels and the ubiquitylation sites on the proteins that were common to both workflows. For this analysis, we only focused on the proteins of human origin. Among the 4,244 quantified proteins of human origin from the global analysis, 128 quantified proteins contained ubiquitylation sites that were quantified in the ubiquitylation workflow (Fig. 6a). In this set of commonly identified proteins, 91 % (116) had unchanged levels of total protein relative abundance. Although there were 10 ubiquitylation sites on 9 proteins with up- or down-regulated relative abundance (6 and 4 ubiquitylation sites, respectively) in the set of commonly identified proteins, the global relative abundance levels of 5 of these proteins were also up- or down-regulated. Consequently, only 4 of the up- or down-regulated ubiquitylation sites were localized to proteins with unchanged relative abundance (Table 1 and Fig. 6b, dashed rectangular box). This underscores the importance of analyzing total protein levels when determining differences in ubiquitylation relative abundance towards the calculation of ubiquitylation site occupancy.

**Table 1:** Proteins of human origin with unchanged total relative abundance levels that have up-regulated (italic text) or down-regulated (bold text) ubiquitylation levels in the luminal vs. basal xenografts

Protein	Accession	Protein function	Total protein relative abundance	Ubiquitylation site (relative abundance)
NADPH-cytochrome P450 reductase [Homo sapiens]	127139033	Electron donation from NADPH to microsomal P450 enzymes	−1.20	**K613 (-2.47)**
bifunctional 3′-phosphoadenosine 5′-phosphosulfate synthase 1 [Homo sapiens]	46094058	Mediation of two steps in sulfate activation pathway	−1.29	**K194 (-2.44)**
ATP-binding cassette sub-family E member 1 [Homo sapiens]	108773782	Transport of molecules across extra- and intra-cellular membranes	−1.19	**K250 (-2.36)**
fatty acid synthase [Homo sapiens]	41872631	Catalysis of palmitate synthesis into long-chain saturated fatty acids	0.80	*K1142 (2.46)*

The down-regulated ubiquitylation sites were on NADPH-cytochrome P450 reductase (K613; mean ubiquitylated peptide RSD_Basal1,Basal2; Luminal1,Luminal2_ = 24 %; cognate global peptide not detected), bifunctional 3′-phosphoadenosine 5′-phosphosulfate synthase 1 (K194; mean ubiquitylated peptide RSD_Basal1,Basal2; Luminal1,Luminal2_ = 12.5 %; cognate global peptide mean RSD_Basal1,Basal2; Luminal1,Luminal2_ = 8 %), and ATP-binding cassette sub-family E member 1 (K250; mean ubiquitylated peptide RSD_Basal1,Basal2; Luminal1,Luminal2_ = 10 %; cognate global peptide mean RSD_Basal1,Basal2; Luminal1,Luminal2_ = 9.5 %). The up-regulated ubiquitylation site was on fatty acid synthase (K1142; mean ubiquitylated peptide RSD_Basal1,Basal2; Luminal1,Luminal2_ = 27 %; cognate global peptide mean RSD_Basal1,Basal2; Luminal1,Luminal2_ = 18 %). It is possible that these differentially regulated ubiquitylation sites reflect differences between the basal and luminal breast cancer subtypes represented in the xenografts used for this study [40]. Additional studies with a larger sample size are required to further elucidate this possibility.

## Conclusions

In summary, we conducted an integrated iTRAQ-based quantitative proteomic analysis that enabled the interrogation of the relationship between global protein and endogenous ubiquitylation relative abundance using basal and luminal breast cancer patient-derived xenograft tissue. Among the proteins that were quantified in their global and ubiquitylated forms, 91 % had unchanged levels of total protein relative abundance in the luminal vs. basal tumor xenografts. Of the proteins with unchanged levels of global relative abundance, 4 % (5) had either up- or down-regulated ubiquitylation relative abundance levels. The proteins with regulated ubiquitylation levels – NADPH-cytochrome P450 reductase, bifunctional 3′-phosphoadenosine 5′-phosphosulfate synthase 1, ATP-binding cassette sub-family E member 1, and fatty acid synthase – could have functional implications in the differentiation between the breast cancer subtypes used for this study.

To date, there have been relatively few quantitative studies of endogenous ubiquitylation in tissue. Although a study using label-free quantification to analyze the ubiquitylated proteome of rat brain tissue was recently published [41], to our knowledge, our study represents the first integrated quantitative analysis of endogenous global protein and ubiquitylation in tissue using iTRAQ labeling. Variations of this approach could entail the use of metabolic stable isotope-labeling by amino acids in cell culture (SILAC)-labeled cell lines as spike-in standards [42, 43] as opposed to chemical-based stable isotope labeling. A primary benefit of using SILAC-labeled cell lines as spike-in standards would be the potentially decreased technical variability because the biological samples could be combined at an early stage in the sample preparation workflow. It has been shown that the error associated with multiple steps of sample preparation in a SILAC-based comparative experiment entailing immunoaffinity enrichment can be limited to a reasonably low level to enable optimal quantitative accuracy [44].

The workflow described in our study permits the analysis of global protein and ubiquitylation site relative abundance toward the determination of ubiquitylation site occupancy. Our observation that 61 % of the quantified ubiquitylation sites were on proteins whose global relative abundance levels were up- or down-regulated underscores the importance of analyzing the modified and unmodified forms of ubiquitylation substrates in studies that are designed to identify ubiquitylation sites that have potential functional significance. Regulated ubiquitylation sites that occur on proteins whose global levels of relative abundance are also up-or down-regulated are sites that are not likely involved in biological information processing. Rather, it is possible that the regulated ubiquitylation sites with functional roles in biological information processing are those that occur on proteins with stable global relative abundance levels.

## Methods

### Chemicals and reagents

Dithiothreitol (DTT), 2-chloroacetamide, Tris-HCl, EDTA, aprotinin, leupeptin, PR-619, PMSF, and LC-MS-grade water were purchased from Sigma-Aldrich (St. Louis, MO). Ultra-pure urea, NaCl, dimethyl pimelimidate, LC-MS grade acetonitrile, trifluoroacetic acid (TFA), formic acid (FA), PepClean C18 spin columns and Empore C18 solid-phase extraction disks were purchased from Thermo Fisher Scientific (Fair Lawn, NJ). Sequencing grade modified trypsin was purchased from Promega (Madison, WI). Sep-Pak SPE C18 cartridges (50 mg, 1 cc) were obtained from Waters (Milford, MA). iTRAQ 4-plex reagent kits were purchased from AB Sciex (Framingham, MA).

### Tissue homogenization

Aliquots of frozen, powderized patient-derived xenograft tumors from established basal (WHIM2, passage 32) and luminal A (WHIM16, passage 33) breast cancer subtypes were obtained from the Washington University Clinical Proteomic Tumor Analysis Consortium (CPTAC) Proteome Characterization Center. The xenografts were raised subcutaneously in 8 week old NOD.Cg-*Prkdc*^*scid*^*Il2rg*^*tm1Wjl*^*/*SzJ mice (Jackson Labs, Bar Harbor, Maine) as previously described [29, 45]. All animal procedures were reviewed and approved by the Institutional Animal Care and Use Committee at Washington University in St. Louis, MO.

Two aliquots of xenograft tumor tissue from basal subtype and two aliquots of xenograft tumor tissue from luminal subtype were used. Tissues were sonicated in denaturing buffer (8 M urea, 50 mM Tris-HCl pH 8, 150 mM NaCl, 1 mM EDTA, 50 μM PR-619, 1 mM 2-chloroacetamide) supplemented with protease inhibitors (2 μg/mL aprotinin, 10 μg/mL leupeptin, 1 mM PMSF). One hundred μL denaturing buffer was added per 10 mg tissue. Lysates were sonicated and cleared by centrifugation at 16,000 x g for 20 min at 4 °C.

### Protein digestion and iTRAQ labeling

Protein concentrations were determined using the bicinchoninic acid (BCA) protein assay (Thermo Fisher Scientific), and 4 mg protein was used for the subsequent steps. Cysteines were reduced with 5 mM dithiothreitol (DTT) for 1 h at 37 °C and alkylated using 10 mM 2-chloroacetamide for 45 min at room temperature (~23 °C) in the dark. The alkylation was conducted using 2-chloroacetamide as opposed to the more commonly used alkylating reagent iodoacetamide to avoid the formation of Lys adducts that are identical in atomic composition to the di-Gly ubiquitin remnant [46]. After 4-fold dilution with 50 mM Tris-HCl, pH 8, trypsin was added at an enzyme-to-substrate ratio of 1:50 (wt/wt), and the digestion was allowed to proceed for 4 h at 37 °C with shaking. An additional aliquot of trypsin was added (1:50; wt/wt) for overnight digestion at 37 °C with shaking. Protease digestion was stopped by the addition of TFA to a final concentration of 1 %, and the digests were subsequently desalted using 50 mg, 1 cc Sep-Pak SPE C18 cartridges (Waters). Sep-Pak SPE C18 cartridges were conditioned with 1 mL 100 % acetonitrile, followed by 1 mL 50 % acetonitrile/0.1 % TFA, and equilibrated with 2 mL 0.1 % TFA. The peptides were loaded onto the conditioned Sep-Pak SPE C18 cartridges, washed with 3 mL 0.1 % TFA and eluted with 0.5 mL 50 % acetonitrile/0.1 % FA. The A_280nm_-based concentration of the de-salted peptide samples was determined using a NanoDrop spectrophotometer (Thermo Fisher Scientific). Aliquots of each de-salted peptide sample (75 μg) were prepared and dried in a SpeedVac concentrator prior to iTRAQ labeling. Three mg aliquots of each de-salted peptide sample were prepared and dried by lyophilization using a vacuum freeze dryer (Labconco) prior to K-ɛ-GG peptide enrichment.

### K-ε-GG peptide enrichment

The anti-K-ε-GG ubiquitin remnant motif antibody (component of the PTMScan® ubiquitin remnant motif kit, Cell Signaling Technology, Danvers, MA) was cross-linked with DMP according to the procedure detailed in [18]. Briefly, the antibody-coupled beads were washed three times with 1 mL of 100 mM sodium bicarbonate, pH 9. The beads were then re-suspended in 1 mL of 20 mM DMP in 100 mM sodium bicarbonate and incubated for 30 min at room temperature with end-over-end rotation. The cross-linking reaction was stopped by washing the beads twice with 1 mL of 200 mM ethanolamine, pH 8 and incubating for 2 h at 4 °C with end-over-end rotation. The cross-linked antibody was washed three times with 1.5 mL of immunoaffinity purification (IAP) buffer (50 mM MOPS pH 7.2, 10 mM sodium phosphate, 50 mM NaCl).

Each dried 3 mg peptide aliquot was reconstituted in 1.5 mL of IAP buffer, and approximately 35 μg of the cross-linked anti-K-ε-GG ubiquitin remnant motif antibody was added to each sample followed by incubation for 1 h at 4 °C with end-over-end rotation. The samples were washed twice with 1.5 mL of IAP buffer followed by three washes with PBS. Peptides were eluted by adding 50 μL of 0.15 % TFA and incubating at room temperature for 5 min. The elution procedure was repeated once and the eluates were combined. Peptides were de-salted using 200 μL C18 Stage Tips [47] packed with three C18 Empore extraction disks. De-salted peptides were dried in a SpeedVac concentrator.

Because the anti-K-ε-GG antibody that was used to enrich for ubiquitin remnant motif-containing peptides does not recognize iTRAQ-labeled peptides, the iTRAQ labeling of the samples for the ubiquitylation analysis was conducted after anti-K-ε-GG enrichment. The iTRAQ labeling scheme was the same as the global samples: 114 (WHIM2 – basal tumor 1: aliquot P32–16), 115 (WHIM2 – basal tumor 2: aliquot P32–17), 116 (WHIM16 – luminal tumor 1: aliquot P33–17), and 117 (WHIM16 – luminal tumor 2: aliquot P33–18). The iTRAQ-labeled, K-ε-GG-enriched samples were combined, de-salted using PepClean C18 spin columns according to the manufacturer’s instructions and dried in a SpeedVac concentrator.

### Basic reversed phase chromatography – global proteome analysis

For the global proteome analysis, 240 μg de-salted peptides were labeled with iTRAQ 4-plex 114 (WHIM2 – basal tumor 1: 60 μg), 115 (WHIM2 – basal tumor 2: 60 μg), 116 (WHIM16 – luminal tumor 1: 60 μg), and 117 (WHIM16 – luminal tumor 2: 60 μg) reagents. Peptides were dissolved in 30 μL of 0.5 M triethylammonium bicarbonate. Seventy μL of ethanol was added to each tube containing 1 U iTRAQ labeling reagent. After the peptides were added to the tubes containing the respective iTRAQ labeling reagent, the labeling reaction was allowed to proceed for 1 h at room temperature. The labeling reaction was terminated by hydrolysis of the iTRAQ reagents with the addition of 300 μL 0.05 % TFA followed by incubation at room temperature for 30 min. The peptides were then mixed, dried in a SpeedVac concentrator and subsequently de-salted using a 50 mg, 1 cc Sep-Pak SPE C18 cartridge and dried again in a SpeedVac concentrator.

Approximately 100 μg of the combined iTRAQ-labeled sample was subjected to offline basic reversed phase liquid chromatography (bRPLC) using a 4.6 x 100 mm Zorbax Extend-1.8 μm C18 column (Agilent Technology, Santa Clara, CA) on an Agilent 1220 Infinity HPLC System. The solvent consisted of 10 mM ammonium formate (pH 10) in water as mobile phase A and 10 mM ammonium formate in 90 % ACN (pH 10) as mobile phase B. The separation gradient was set as follows: 2 % B for 10 min, 2 – 8 % B for 5 min, 8 – 35 % B for 85 min, 35 – 95 % B for 5 min, and 95 % B for 25 min. The 96 fractions were pooled in a non-contiguous manner into 24 fractions by combining fractions 1, 25, 49, and 73; 2, 26, 50, and 75, etc. The concatenated fractions were dried in a SpeedVac concentrator.

### LC-MS/MS analysis

Peptides were resuspended in LC solvent A (2 % acetonitrile/0.1 % formic acid) and analyzed by nanoflow LC-MS/MS using an LTQ-Orbitrap Velos Pro mass spectrometer (Thermo Fisher Scientific) coupled online to a Dionex Ultimate 3000 RSLCnano system (Thermo Fisher Scientific) with a 75 μm x 50 cm Acclaim PepMap RSLC 2 μm C18 separating column (Thermo Fisher Scientific) protected by a 100 μm x 2 cm Acclaim PepMap100 5 μm C18 guard column (Thermo Fisher Scientific). The mobile phase flow rate was 300 nL/min and consisted of 0.1 % formic acid in water (A) and 0.1 % formic acid in 95 % acetonitrile (B). The gradient profile was set as follows: 2 – 22 % B for 70 min, 22 – 29 % B for 8 min, 29 – 95 % B for 4 min, and 95 % B for 8 min. The mass spectrometer spray voltage was set at 2.2 kV. Full scan Orbitrap spectra (AGC 1x10^6^) were collected from 400–1800 m/z at a resolution of 30,000 with a maximum injection time of 10 ms followed by data-dependent HCD MS/MS (7,500 resolution, 35 % collision energy, 0.1 ms activation time, 100 ms maximum injection time, 1x10^4^ AGC) of the 10 most abundant ions using an isolation width of 2.0 Th. The minimum signal required to trigger an MS2 scan was 500, and the first mass value was fixed at 100 m/z. Charge state screening was enabled to reject the acquisition of MS/MS spectra for unassigned and singly charged precursor ions. A dynamic exclusion duration of 25 s was used.

### MS data analysis

MS/MS spectra were searched with SEQUEST HT using Proteome Discoverer version 1.4 (Thermo Fisher) against a customized combined NCBI RefSeq Release 37 of *Homo sapiens* and *Mus musculus* database with 55,415 entries (http://fenchurch.mc.vanderbilt.edu/misc/20111201-RefSeq-Human-37-Mouse-37-Trypsin.fasta). The precursor and fragment mass tolerances were set to 20 ppm and 0.05 Da, respectively. Enzymatic cleavage specificity was set to fully tryptic with three missed cleavages allowed. For the global proteomic dataset, carbamidomethylation of cysteine and iTRAQ 4-plex modification of peptide N-termini were set as fixed modifications, and oxidation of methionine and iTRAQ 4-plex modification of Lys were set as variable modifications. The data were also searched against a decoy database (reversed protein sequences) and filtered using a 1 % false discovery rate. Only peptides with a search engine rank of 1 and protein groups identified by at least two unique (non-redundant) peptide sequences were considered. Protein ratios were normalized based on the mean ratio distribution of each dataset.

For the ubiquitylation dataset, to account for the additional amine group that is present on the –GG remnant of the ubiquitylated peptides that could be labeled by the iTRAQ reagent, a variable modification of +258.1449 Da on Lys (mass of iTRAQ 4-plex reagent and two Gly residues) was included. GlyGly addition to Lys was also used as a variable modification. Four modifications per peptide were permitted. The data were also searched against a decoy database (reversed protein sequences) and filtered with a 1 % false discovery rate. Only peptides with a search engine rank of 1 were considered. All spectra that were assigned to ubiquitylated peptides were manually validated. Di-Gly-modified Lys residues located on peptide C-termini were considered false positive identifications and were removed.

Peptide and protein quantification was based on the iTRAQ reporter ion intensities using Proteome Discoverer v.1.4. Only unique (non-redundant) peptide sequences were used for protein quantification. Although methionine residues are chemically unstable and prone to oxidation, peptides containing these amino acids (<1 % of the ubiquitylated peptides in our dataset) were not excluded from quantification. Unless otherwise noted, all quantification data refer to only peptides and proteins of human origin. Ubiquitylated peptide normalization was based on the mean ratio distribution of each dataset. Ubiquitylation sites or proteins with relative abundance ratios greater or less than the mean ± 2 s.d. of the distribution of ratios for each dataset were considered to be up- or down-regulated, respectively, in the luminal compared to the basal breast cancer tumor xenografts.

### Gene ontology functional analysis

STRAP (Software for Researching Annotations of Proteins) [35] was used to determine the distribution of Gene Ontology cellular compartment terms in the ubiquitylation dataset.

## Additional files

Additional file 1: Table S1. List of quantified ubiquitylated peptides from basal and luminal breast tumor xenografts subjected to anti-di-Gly ubiquitin remnant immunoaffinity enrichment. The log2 peptide ratio from each replicate is an average of all the quantified peptide-spectrum matches for a given peptide. CVs are reported for peptides quantified by >1 peptide-spectrum match. Peptides from human and murine proteins are included. Additional file 2: Table S2. List of quantified proteins from global proteome analysis of basal and luminal breast tumor xenografts. Proteins of human and murine origin are included.

## Abbreviations

K-ε-GG: Di-glycine remnant
MS: Mass spectrometry
GO: Gene ontology
bRPLC: Basic reversed phase liquid chromatography
